# Comparative Effects of Tumor Necrosis Factor Alpha, Lipopolysaccharide, and Palmitate on Mitochondrial Dysfunction in Cultured 3T3-L1 Adipocytes

**DOI:** 10.1007/s12013-024-01522-3

**Published:** 2024-09-13

**Authors:** Babalwa Unice Jack, Stephanie Dias, Carmen Pheiffer

**Affiliations:** 1 Biomedical Research and Innovation Platform, South African Medical Research Council, Tygerberg, Cape Town, 7505 South Africa; 2 Centre for Cardiometabolic Research in Africa, Division of Medical Physiology, Stellenbosch University, Tygerberg, Cape Town, 7505 South Africa; 3 Department of Obstetrics and Gynaecology, Faculty of Health Sciences, University of Pretoria, Pretoria, 0001 South Africa

**Keywords:** Obesity, 3T3-L1 adipocytes, Mitochondrial dysfunction, TNFα, LPS, Palmitate

## Abstract

We have previously reported that dysregulated lipid metabolism and inflammation in 3T3-L1 adipocytes is attributed to tumor necrosis factor alpha (TNFα) rather than lipopolysaccharide (LPS) and palmitate (PA). In this study, we further compared the modulative effects of TNFα, LPS, and PA on mitochondrial function by treating 3T3-L1 adipocytes with TNFα (10 ng/mL), LPS (100 ng/mL), and PA (0.75 mM) individually or in combination for 24 h. Results showed a significant reduction in intracellular adenosine triphosphate (ATP) content, mitochondrial bioenergetics, total antioxidant capacity, and the mRNA expression of citrate synthase (*Cs*), sirtuin 3 (*Sirt3*), protein kinase AMP-activated catalytic subunit alpha 2 (*Prkaa2*), peroxisome proliferator-activated receptor gamma coactivator 1 alpha (*Ppargc1α*), nuclear respiratory factor 1 (*Nrf1*), and superoxide dismutase 1 (*Sod1*) in cells treated with TNFα individually or in combination with LPS and PA. Additionally, TNFα treatments decreased insulin receptor substrate 1 (*Irs1*), insulin receptor substrate 2 (*Irs2*), solute carrier family 2, facilitated glucose transporter member 4 (*Slc2a4*), and phosphoinositide 3 kinase regulatory subunit 1 (*Pik3r1*) mRNA expression. Treatment with LPS and PA alone, or in combination, did not affect the assessed metabolic parameters, while the combination of LPS and PA increased lipid peroxidation. These results show that TNFα but not LPS and PA dysregulate mitochondrial function, thus inducing oxidative stress and impaired insulin signaling in 3T3-L1 adipocytes. This suggests that TNFα treatment can be used as a basic in vitro model for studying the pathophysiology of mitochondrial dysfunction and related metabolic complications and screening potential anti-obesity therapeutics in 3T3-L1 adipocytes.

## Introduction

Over the years, the prevalence of obesity has continued to increase at an alarming rate, placing a severe burden on individuals, the public, and healthcare systems [1]. Obesity is progressively being linked to an increased risk of developing obesity-related chronic diseases, including non-alcoholic fatty liver disease (NAFLD), type 2 diabetes (T2D), cardiovascular disease (CVD), and several types of cancers [2]. Although not fully elucidated, various studies point to adipocyte dysfunction as a significant contributor to the pathophysiology of obesity-related chronic disease [3, 4]. As a result, research on obesity has reignited interest in studying adipose tissue to elucidate the pathophysiological mechanisms that link obesity to these chronic diseases and to establish adipose tissue as a potential target for anti-obesity therapeutic agents [5]. Previously considered to be an inert tissue for the storage and release of energy during nutrient surplus and deficit, adipose tissue is now recognized as an endocrine organ that modulates energy homeostasis [6]. Adipose tissue is known to undergo adverse alterations during obesity that predispose to metabolic dysregulation, including low-grade chronic inflammation and insulin resistance [7, 8]. This induces adipocyte dysfunction, which is characterized by high levels of pro-inflammatory cytokines such as tumor necrosis factor alpha (TNFα) [9], increased levels of free fatty acids (FFAs), particularly saturated FFAs such as palmitate (PA) [10], and elevated levels of bacterial-derived endotoxins such as lipopolysaccharide (LPS) [11].

TNFα is a cytokine primarily known for its pro-inflammatory effects and has been implicated in various inflammatory diseases. Clinical studies have shown that elevated systemic levels of TNFα as well as overexpression of TNFα in adipose tissue are associated with obesity, insulin resistance, and T2D [12–14]. Further, in experimental models of obesity and diabetes, TNFα is abnormally high in adipose tissues and is associated with insulin resistance [15]. TNFα induces insulin resistance by directly inhibiting the insulin signaling pathway, modulating mitochondrial oxidative phosphorylation (OxPhos), and inducing oxidative stress signaling pathways [16]. In obese individuals, the circulating levels of FFAs such as PA are high due to increased FFA release associated with adipose tissue dysfunction, and this exacerbates insulin resistance [17]. Studies have shown that PA leads to insulin resistance by directly impairing the insulin signaling pathway or inducing lipotoxicity, mitochondrial dysfunction, and inflammation [18, 19]. In addition, obesity is associated with increased circulating levels of LPS, which triggers insulin resistance by inducing inflammatory signals that interfere with insulin signaling [20]. This has been demonstrated in high-fat diet-induced mice with elevated systemic LPS concentration and increased fasting glucose and insulin levels [21]. Mitochondrial OxPhos plays an important role in generating the bulk of cellular adenosine triphosphate (ATP), via a process that transports electrons through the five multimeric complexes entrenched in the inner mitochondrial membrane to generate a proton gradient for ATP synthesis [22]. In dysfunctional adipocytes, mitochondrial OxPhos is defective and induces mitochondrial dysfunction via excessive mitochondrial reactive oxygen species (ROS) production [23, 24]. The overproduction of mitochondrial ROS, which is the case in dysfunctional adipocytes, induces oxidative stress, subsequently contributing to the development of insulin resistance [25]. This highlights the important role of TNFα, LPS, and PA in the pathophysiology of adipocyte dysfunction.

Our previous study compared the effects of TNFα, LPS, and PA on lipid metabolism and inflammation in murine 3T3-L1 adipocytes. We reported that TNFα stimulates lipolysis and pro-inflammatory responses while reducing lipid content and anti-inflammatory responses [26]. Meanwhile, treatment with LPS and PA individually or in combination had no effect [26]. Our study also showed that treatment with the combination of TNFα, LPS, and PA had similar effects as TNFα individually, thus suggesting that TNFα is sufficient to mimic an in vitro model of adipocyte dysfunction characterized by dysregulated lipid metabolism and increased inflammation [26]. Although TNFα has been shown to modulate lipid metabolism and inflammation in these cells, its role either individually or in combination with LPS and PA on mitochondrial function has not been fully explored. Hence, in this study, we aimed to conduct a comparative analysis of the effects of TNFα, LPS, and PA or their combination effects on mitochondrial dysfunction and associated metabolic complications including oxidative stress damage and insulin resistance in cultured 3T3-L1 adipocytes.

## Materials and Methods

### Materials and Reagents

3T3-L1 mouse embryonic fibroblasts (catalog number CL-173) were bought from the American Type Culture Collection (Manassas, VA, United States). Dulbecco’s Modified Eagle Medium (DMEM), Dulbecco’s Phosphate Buffered Saline (DPBS), L-Glutamine, Sodium Pyruvate, and the ViaLight^TM^ Plus Cell Proliferation and Cytotoxicity BioAssay Kit were purchased from Lonza (Walkersville, MD, United States). Fetal Bovine Serum (FBS) was obtained from Gibco (Thermo Fisher Scientific, Waltham, MA, United States). Bovine Serum Albumin Fraction V Fatty Acid-Free (FAF-BSA) was from Roche (Mannheim, Germany). Mouse Tumor Necrosis Factor Alpha (TNFα), Lipopolysaccharide (LPS, *Escherichia coli* O55:B5), Palmitate (PA), 3-Isobutyl-1-Methylxanthine (IBMX), Insulin Human Solution, Dexamethasone, D-Glucose Solution (45% in water), Sodium Bicarbonate (NaHCO_3_), and the Antioxidant assay kit were obtained from Sigma-Aldrich (St. Louis, MO, United States). The OxiSelect™ Thiobarbituric Acid reactive substances (TBARS) assay kit was purchased from Cell Biolabs (San Diego, CA, United States). The Reducing Agent Compatible and Detergent Compatible (RC DC) protein assay kit and Bovine Serum Albumin (BSA) standards were obtained from Bio-Rad Laboratories (Hercules, CA, United States). Seahorse XF Cell Mito Stress test kit, XF96 Cell Culture Microplates, XFe96 Sensor Cartridges, and XF Base Medium were supplied by Agilent (Santa Clara, CA, United States). QIAzol Reagent, RNeasy Mini kit, and the AllPrep DNA/RNA/Protein Mini kit were supplied by Qiagen (Hilden, Germany). All other chemicals were obtained from Sigma-Aldrich, or otherwise stated.

### Cell Culture, Differentiation, and Treatment

3T3-L1 mouse embryonic fibroblasts were cultured in growth medium (DMEM supplemented with 10% FBS) at 37 °C in humidified air with 5% CO_2_ and subsequently sub-cultured every 2 to 3 days. For experimental purposes, 3T3-L1 pre-adipocytes were seeded in multi-well plates at a seeding density of 2 × 10^4^ cells/mL, and once fully confluent (day 0), cells were induced to differentiate into mature adipocytes as previously described [26–29]. Briefly, the growth medium was replaced with adipocyte differentiation medium (DMEM supplemented with 10% FBS, 0.5 mM IBMX, 1 μg/mL insulin, and 1 μM dexamethasone) for 72 h. At day 3, the adipocyte differentiation medium was changed to adipocyte maintenance medium (DMEM supplemented with 10% FBS and 1 µg/mL insulin), and the cells were incubated for a further 48 h. At day 5, the differentiated adipocytes were cultured in a growth medium that was refreshed daily until cells became fully differentiated. At day 8, fully differentiated 3T3-L1 adipocytes were washed with DPBS and treated for 24 h with TNFα (10 ng/mL), LPS (100 ng/mL), and PA (0.75 mM) either alone or in various combinations as reported previously [26].

### Metabolic Activity

Intracellular ATP levels were quantified using the ViaLight^TM^ Plus Cell Proliferation and Cytotoxicity BioAssay kit as per the manufacturer’s instructions with minor modifications. After treatment, 3T3-L1 adipocytes were washed with pre-warmed DPBS and allowed to cool at room temperature, followed by lysing the cells for at least 10 min at room temperature using an ATP cell lysis reagent. Following incubation, the cell lysate was transferred to a luminescence-compatible white 96 well plate and incubated with a reconstituted ATP monitoring reagent plus for 2 min before luminescence was measured on a SpectraMax^®^ i3x Multi-Mode Microplate reader using the SoftMax Pro 7 Software (Molecular Devices, San Jose, CA, United States). Results for intracellular ATP levels were expressed as a percentage relative to the control.

### Mitochondrial Bioenergetics

The bioenergetic profiles of 3T3-L1 adipocytes, including oxygen consumption rate (OCR) and extracellular acidification rate (ECAR), were measured using the XF96 extracellular flux analyzer (Agilent) as described previously with minor modifications [30, 31]. In brief, 3T3-L1 pre-adipocytes were seeded in XF96 microplates (10 000 cells per 80 μL per well) to reach confluency after 24 h and subsequently differentiated into adipocytes as described above. On day 5, differentiated 3T3-L1 adipocytes were treated as described above. Following treatment, cells were washed with pre-warmed XF base assay medium supplemented with 25 mM glucose, 2 mM glutamine, and 1 mM pyruvate and thereafter incubated in 180 μL XF base assay medium for 15 min at 37 °C in a non-CO_2_ incubator to equilibrate temperature and pH before OCR and ECAR measurements. After 15 min, the XF Cell Mito Stress test kit was used to measure the key parameters of mitochondrial respiration including basal respiration, ATP production, maximal respiration, and spare respiratory capacity by directly measuring mitochondrial OCR over a time course before and after injecting with oligomycin (ATP-synthase inhibitor, 2 μM), carbonyl cyanide p-trifluoromethoxyphenylhydrazone (FCCP, a mitochondrial uncoupler, 0.75 μM), and rotenone/antimycin A (complex I and complex III inhibitors, respectively, 0.5 μM). After the assay, the cells were used to determine protein concentration using the RC DC protein assay kit as per the manufacturer’s instructions, and data was normalized relative to the total protein content and represented as pmol/min/µg protein for OCR and mpH/min/µg protein for ECAR.

### Mitochondrial DNA Content

Genomic DNA was extracted from 3T3-L1 adipocytes after 24 h treatment using the AllPrep^®^ DNA/RNA/Protein Mini Kit as per manufacturer’s instructions. DNA concentrations were measured using the NanoDrop™ One/One^c^ microvolume UV-Vis Spectrophotometer (Thermo Fisher Scientific). To quantify mitochondrial DNA (mtDNA) copy numbers, 20 ng of genomic DNA was subjected to quantitative real-time PCR (qRT-PCR) using Taqman^®^ gene expression assays (Applied Biosystems, Foster City, CA, United States) specific for the mitochondrial NADH dehydrogenase subunit 1 (*Nd1*) and the nuclear actin beta (*Actb*) genes (Table 1). PCR reactions were run on the QuantStudio™ 7 Flex Real-Time PCR System (Applied Biosystems) using the standard PCR conditions for Taqman probes. The number of copies of mtDNA was calculated using the 2 × 2^ΔCt^ method, where delta Ct (ΔCt) was calculated as the difference in Ct values between *Actb* and *Nd1*, and results were expressed as relative mtDNA content as previously reported [32].

**Table 1:** Taqman gene expression assays

Taqman gene expression assays	Assay ID	Role
Protein kinase AMP-activated catalytic subunit alpha 2 (*Prkaa2*/*Ampkα2*)	Mm01264789_m1	Cellular energy metabolism
Citrate synthase (*Cs*)	Mm00466043_m1	Mitochondrial content and function
Sirtuin 3 (*Sirt3*)	Mm00452131_m1	Mitochondrial function
Peroxisome proliferator-activated receptor gamma coactivator 1 alpha (*Ppargc1a*)	Mm01208835_m1	Mitochondrial biogenesis
Nuclear respiratory factor 1 (*Nrf1*)	Mm01135606_m1	Mitochondrial biogenesis
Superoxide dismutase 1 (*Sod1*)	Mm01344233_g1	Antioxidant enzyme
DNA-damage inducible transcript 3 (*Ddit3*/*Chop*)	Mm00492097_m1	Endoplasmic reticulum stress
Heat shock protein family A (Hsp70) member 5 (*Hspa5*/*Bip*/*Grp78*)	Mm00517691_m1	Endoplasmic reticulum stress
X-box binding protein 1 (*Xbp1*)	Mm00457357_m1	Endoplasmic reticulum stress
Insulin receptor substrate 1 (*Irs1*)	Mm01278327_m1	Insulin signaling
Insulin receptor substrate 2 (*Irs2*)	Mm03038438_m1	Insulin signaling
Phosphoinositide 3 kinase regulatory subunit 1 (*Pik3r1*)	Mm00803160_m1	Insulin signaling
Solute carrier family 2, facilitated glucose transporter member 4 (*Slc2a4*/*Glut4*)	Mm01245502_m1	Glucose transport
NADH dehydrogenase subunit 1 (*Nd1*)	Mm04225274_s1	Mitochondrial gene (mtDNA content)
Actin, beta (*Actb*)	Mm04394036_g1	Nuclear gene (mtDNA content)
Beta 2 microglobulin (*B2m*)	Mm00437762_m1	Housekeeping gene
Ribosomal protein L13a (*Rpl13a*)	Mm01612986_gH	Housekeeping gene

### Lipid Peroxidation

Lipid peroxidation was assessed by quantifying malondialdehyde (MDA) content using the OxiSelect™ thiobarbituric acid (TBA) reactive substances (TBARS) assay kit. After treatment, the media was removed, and cells were washed with pre-warmed DPBS. Thereafter approximately 1 × 10^7^ cells/mL were collected in PBS supplemented with 0.05% butylated hydroxytoluene solution (to prevent oxidation) and homogenized on ice for 1 min (repeated three times) at 25 Hz using a Tissue lyser and pre-cooled adapters (Qiagen). Subsequently, 100 µL of the homogenates and MDA standards were thoroughly mixed with 100 µL of sodium dodecyl sulfate lysis solution and incubated for 5 min at room temperature. Thereafter, 250 µL of TBA reagent was added to the homogenates and standards, and the samples were incubated at 95 °C for 60 min, cooled to room temperature in an ice bath for 5 min, followed by centrifuge at 3,000 rpm for 15 min. Thereafter, MDA content from the supernatants was quantified by spectrophotometric measurements at an absorbance of 570 nm using the BioTek^®^ ELx800 microplate reader and Agilent BioTek Gen5^®^ Imaging Software for data acquisition (BioTek Instruments Inc., Winooski, VT, United States). The MDA content of the test samples was derived from the standard curve and the results were expressed as µM MDA content.

### Total Antioxidant Capacity

The total antioxidant capacity (TAC) was measured using a colorimetric antioxidant assay kit as per the manufacturer’s instructions. Briefly, 3T3-L1 adipocytes were washed with pre-warmed DPBS after treatment, and the cells (approximately 1 × 10^6^) were collected in a cold 1× assay buffer, homogenized on ice for 1 min (repeated three times) at 25 Hz using a Tissue lyser and pre-cooled adapters (Qiagen), and then centrifuged at 12,000 × g for 15 min at 4 °C. Thereafter, 10 µL of the supernatant of each sample and the Trolox standards, and 20 µL of the myoglobin working solution were incubated with 150 µL of ABTS (2,2’-azino-bis(3-ethylbenzothiazoline-6-sulfonic acid)) substrate working solution for 10 min at room temperature. After incubation, 100 µL of stop solution was added to the test samples and Trolox standards, and absorbance was read by measuring optical density (OD) at 405 nm using the BioTek^®^ ELx800 microplate reader equipped with Agilent BioTek Gen5^®^ Imaging Software for data acquisition (BioTek Instruments Inc.). The antioxidant concentration of the test samples was calculated using the equation obtained from the linear regression of the standard curve and the results were expressed as mM Total Antioxidant Capacity.

### mRNA Gene Expression

Quantitative analysis of messenger RNA (mRNA) expression was performed as previously described [26]. Following total RNA isolation and purification, complementary DNA (cDNA) was synthesized using the High-Capacity Reverse Transcription kit, based on the manufacturer’s instructions (Applied Biosystems). The cDNA was then amplified by qRT-PCR using the TaqMan™ fast advanced master mix and Taqman^®^ gene expression assays (Table 1) on a QuantStudio™ 7 Flex Real-Time PCR System (Applied Biosystems). The relative standard curve quantification method was used to quantify the transcription levels which were normalized to an average of two housekeeping genes (Table 1).

### Statistical Analysis

Experimental data are shown as the mean ± standard error of the mean (SEM) of three independent experiments. Statistical analysis was conducted using Graph Pad Prism software (Graph Pad Software Inc. V7.03, San Diego, CA, United States). Significant differences between treatments were determined by the non-parametric Kruskal-Wallis test, followed by Dunn’s multiple comparisons post-test for data that were not normally distributed, as determined by the Shapiro-Wilk test. One-way analysis of variance (ANOVA) and Dunnett’s multiple comparisons post-test was used for data that were normally distributed. All statistical tests with p < 0.05, were considered significant.

## Results

### TNFα Reduces the Metabolic Activity in 3T3-L1 Adipocytes

**Fig. 1:**
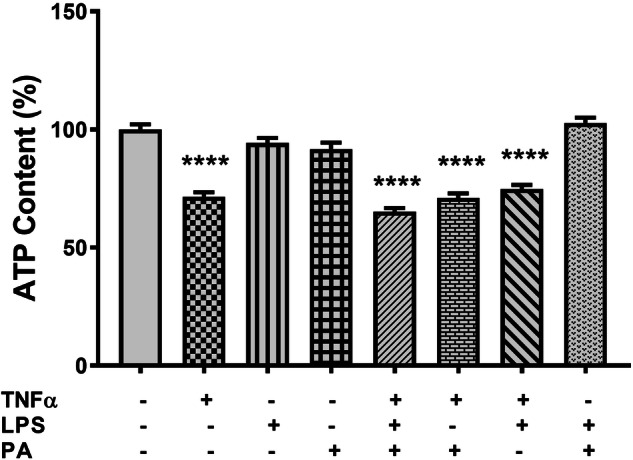
TNFα reduces metabolic activity in 3T3-L1 adipocytes. Differentiated 3T3-L1 adipocytes were treated with either TNFα (10 ng/mL), LPS (100 ng/mL), or PA (0.75 mM) alone or in combination for 24 h. Metabolic activity was assessed by measuring ATP content using a commercial assay kit. Results are expressed as the percentage relative to the control (set at 100%) and are shown as mean ± standard error of the mean (SEM) for three independent biological experimental repeats. Statistical significance is depicted as ****p < 0.0001 compared to the untreated control cells

The effect of TNFα, LPS, and PA on cell metabolic activity was assessed by quantifying intracellular ATP content. Compared to the control (100 ± 10.6%), treatment with TNFα reduced ATP content by 28.5% (71.5 ± 9.1%, p < 0.0001), while LPS or PA had no effect on ATP content (Fig. 1). Furthermore, ATP content was reduced by 34.8% (65.2 ± 7.6%, p < 0.0001), 30% (71.0 ± 9.4%, p < 0.0001), and 25.2% (74.8 ± 8.4%, p < 0.0001) in cells treated with the combination of either TNFα, LPS, and PA, or TNFα and PA, or TNFα and LPS, respectively (Fig. 1). No difference was observed in the ATP content of 3T3-L1 adipocytes when comparing TNFα only treated cells with the TNFα combination treatments. Treatment with the combination of LPS and PA did not affect ATP content (Fig. 1).

### TNFα in Combination with LPS and PA Impairs Mitochondrial Respiratory Function in 3T3-L1 Adipocytes

**Fig. 2:**
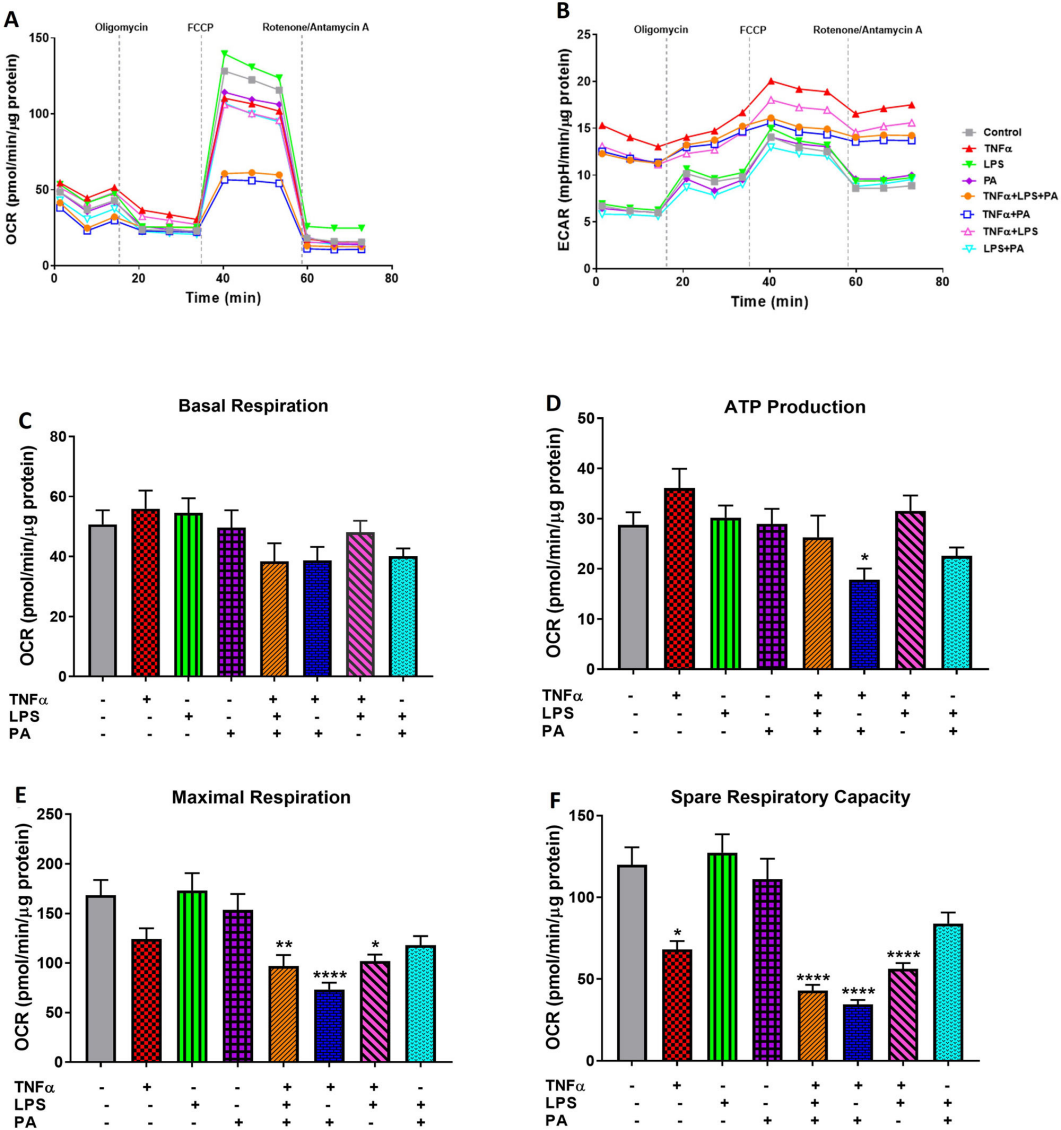
TNFα in combination with LPS and PA impairs mitochondrial respiratory function in 3T3-L1 adipocytes. Differentiated 3T3-L1 adipocytes were treated with either TNFα (10 ng/mL), LPS (100 ng/mL), and PA (0.75 mM) alone or in combination for 24 h. Mitochondrial oxygen consumption rate (OCR) (**A**), extracellular acidification rate (ECAR) (**B**), basal respiration (**C**), ATP production (**D**), maximal respiration (**E**), and spare respiratory capacity (**F**) were determined using the XF96 extracellular flux analyzer. Results are expressed as pmol/min (OCR) and mpH/min (ECAR) and normalized to total protein content (µg). Data are shown as mean ± SEM for three independent biological experimental repeats. Statistical significance is depicted as *p < 0.05, **p < 0.01, and ****p < 0.0001 compared to the untreated control cells

The effects of TNFα, LPS, and PA alone or in combination on mitochondria bioenergetics were assessed by measuring the mitochondrial respiratory capacity (OCR) and the glycolytic rate (ECAR) using the Agilent Seahorse XF Technology. Cells treated with TNFα alone or the combination of TNFα, LPS, and PA, or TNFα and PA, or TNFα and LPS had lower OCR and higher glycolytic activity compared to cells treated with the control, LPS, PA, or the combination of LPS and PA (Fig. 2A, B). Through this, the key parameters involved in mitochondrial respiration including basal respiration, ATP production, maximal respiration, and spare respiratory capacity were measured in the presence of inhibitors. Although not statistically significant, basal mitochondrial respiration was reduced by treatment with the combination of TNFα, LPS, and PA (24.4%, 38.3 ± 6.1 pmol/min/µg protein), TNFα and PA (23.7%, 38.7 ± 4.5 pmol/min/µg protein), and LPS and PA (21.0%, 40.1 ± 2.6 pmol/min/µg protein) when compared to the control (50.7 ± 4.7 pmol/min/µg protein) (Fig. 2C). Following injection with oligomycin, an ATP synthase inhibitor, mitochondrial ATP production was increased by 25.3% in cells treated with TNFα only (36.1 ± 3.9 pmol/min/µg protein), but this was not statistically significant when compared to the control (28.8 ± 2.5 pmol/min/µg protein) (Fig. 2D). In contrast, mitochondrial ATP production was reduced by 38.2% (17.8 ± 2.3 pmol/min/µg protein, p < 0.05) in cells treated with the combination of TNFα and PA while other treatment conditions had no significant effects on mitochondrial ATP production (Fig. 2D).

After injection with the uncoupler, FCCP, reduced maximal respiration in adipocytes treated with TNFα alone (26.3% decrease, 124.1 ± 10.8 pmol/min/µg protein) or the combination of LPS and PA (29.9% decrease, 118.0 ± 9.2 pmol/min/µg protein) was observed, but this was not statistically significant compared to the control cells (168.4 ± 15.3 pmol/min/µg protein) (Fig. 2E). Meanwhile, maximal respiration was significantly reduced by 42.5%, 56.5%, and 39.5%, in cells treated with the combination of either TNFα, LPS, and PA (96.9 ± 11.2 pmol/min/µg protein, p < 0.01), TNFα and PA (73.2 ± 6.9 pmol/min/µg protein, p < 0.0001), and TNFα and LPS (101.8 ± 6.7 pmol/min/µg protein, p < 0.05) when compared to control adipocytes (Fig. 2E). Similarly, after Rotenone/Antamycin A injection, spare respiratory capacity was reduced by 43.2%, 64.3%, 71.2%, 53.0%, and 30.0% in cells treated with TNFα alone (68.2 ± 5.1 pmol/min/µg protein, p < 0.05) or the combinations of either TNFα, LPS, and PA (42.9 ± 3.6 pmol/min/µg protein, p < 0.0001), TNFα and PA (34.5 ± 2.8 pmol/min/µg protein, p < 0.0001), TNFα and LPS (56.4 ± 3.5 pmol/min/µg protein, p < 0.0001), or LPS and PA (84.0 ± 6.7 pmol/min/µg protein, p > 0.05) when compared to control cells (120.0 ± 10.5 pmol/min/µg protein) (Fig. 2F).

### TNFα Decreases mRNA Expression of Genes Involved in Mitochondrial Biogenesis in 3T3-L1 Adipocytes

**Fig. 3:**
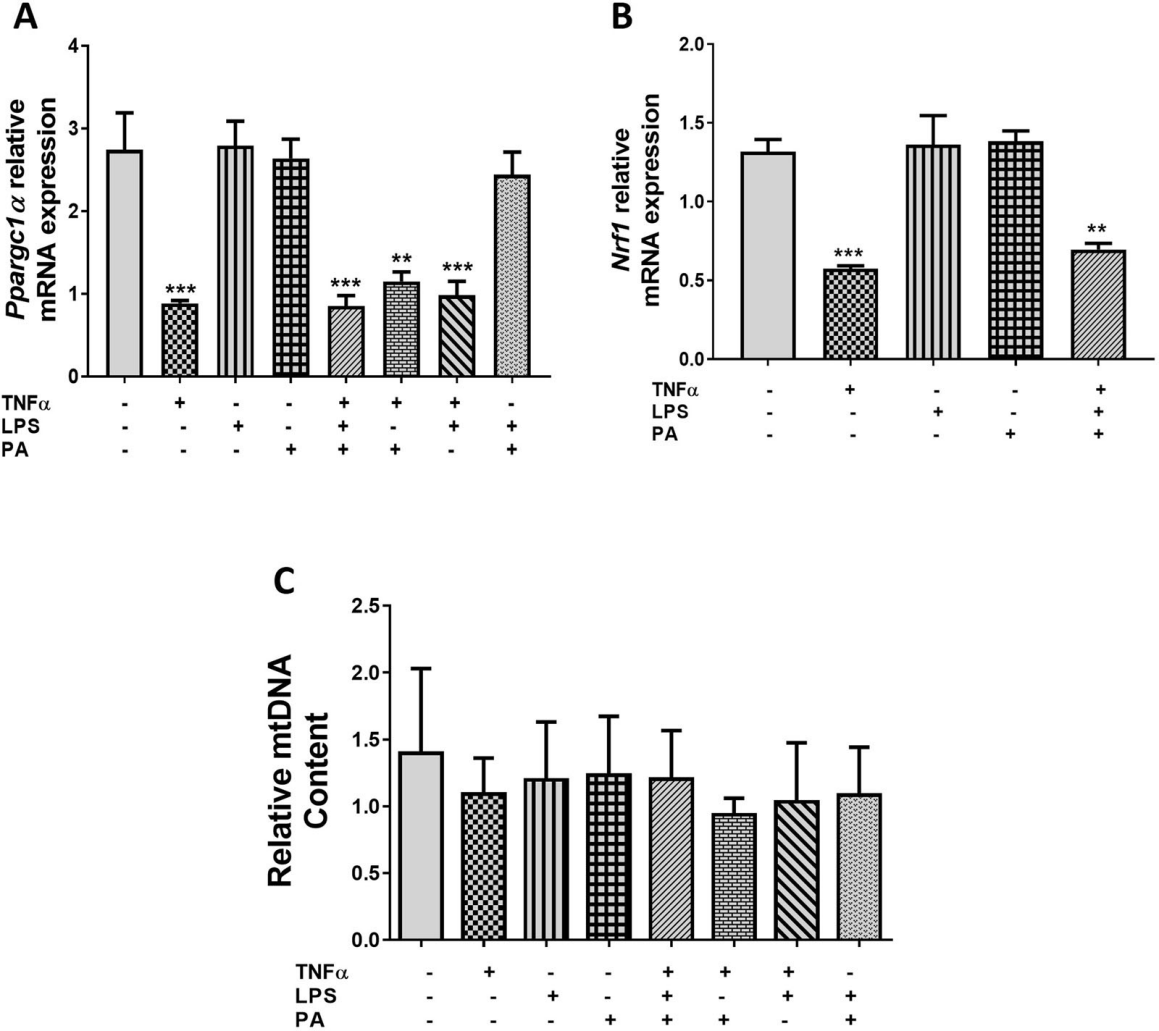
TNFα decreases *Ppargc1α* and *Nrf1* mRNA expression in 3T3-L1 adipocytes. Differentiated 3T3-L1 adipocytes were treated with TNFα (10 ng/mL), LPS (100 ng/mL), and PA (0.75 mM) alone or in combination for 24 h. The relative mRNA expression of *Ppargc1α* (**A**) and *Nrf1* (**B**), and mitochondrial DNA (mtDNA) content (**C**), were measured using qRT-PCR. Results for *Ppargc1α* and *Nrf1* mRNA are expressed relative to the average of *B2m* and *Rpl13a* as arbitrary units, while the number of copies of mtDNA was expressed as relative mtDNA content. Data are shown as the mean ± SEM of three independent biological experiments, each conducted in triplicate. Statistical significance between groups is denoted as **p < 0.01 and ***p < 0.001 compared to the untreated control adipocytes

The effects of TNFα, LPS, and PA alone or in combination on mitochondria biogenesis were assessed by quantifying mitochondrial DNA content and the relative mRNA expression levels of peroxisome proliferator-activated receptor gamma coactivator 1 alpha (*Ppargc1α*) and nuclear respiratory factor 1 (*Nrf1*), key genes involved in mitochondrial biogenesis, using qRT-PCR. Compared to control cells (2.74 ± 0.45), the relative mRNA expression of *Ppargc1α* was reduced 3.1-fold (0.89 ± 0.03, p < 0.001), 3.2-fold (0.85 ± 0.12, p < 0.001), 2.4-fold (1.15 ± 0.11, p < 0.01) and 2.8-fold (0.98 ± 0.17, p < 0.001) in cells treated with either TNFα only, TNFα, LPS, and PA combination, TNFα and PA combination, or TNFα and LPS combination, respectively (Fig. 3A). Similarly, *Nrf1* mRNA expression was also reduced 2.3-fold (0.57 ± 0.02, p < 0.001) and 1.9-fold (0.70 ± 0.04, p < 0.01) in adipocytes treated with TNFα only or the combination of TNFα, LPS, and PA when compared to control adipocytes (1.32 ± 0.08) (Fig. 3B). No difference was observed in the mRNA expression of *Ppargc1α* and *Nrf1* when comparing cells treated with TNFα only and cells treated with the combination of TNFα, LPS, and PA (Fig. 3A, B). In contrast, treatment with TNFα, LPS, and PA alone or in combination did not affect mitochondrial DNA content (Fig. 3C).

### TNFα Reduces the mRNA Expression Levels of Genes Regulating Mitochondrial Function and Energy Metabolism in 3T3-L1 Adipocytes

**Fig. 4:**
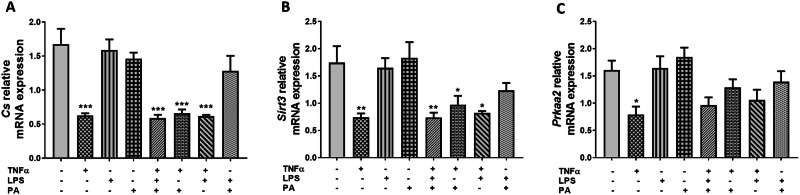
TNFα lowers the mRNA expression levels of genes regulating mitochondrial function and cellular energy metabolism in 3T3-L1 adipocytes. Differentiated 3T3-L1 adipocytes were treated with TNFα (10 ng/mL), LPS (100 ng/mL), and PA (0.75 mM) individually or in combination for 24 h. The relative mRNA expression levels of *Cs* (**A**), *Sirt3* (**B**), and *Prkaa2* (**C**) were measured using qRT-PCR. Results for *Cs*, *Sirt3*, and *Prkaa2* mRNA are expressed relative to the average of *B2m* and *Rpl13a* as arbitrary units. Data are shown as the mean ± SEM of three independent biological experiments, each conducted in triplicate. Statistical significance between groups is denoted as *p < 0.05, **p < 0.01, and ***p < 0.001 compared to the untreated control adipocytes

The relative mRNA expression levels of citrate synthase (*Cs*), sirtuin 3 (*Sirt3*), and protein kinase AMP-activated catalytic subunit alpha 2 (*Prkaa2*), key genes involved in mitochondrial function and cellular energy homeostasis were measured by qRT-PCR in response to treatment with TNFα, LPS, and PA alone or in combination. Compared to control adipocytes (1.67 ± 0.23), the relative mRNA expression of *Cs* was reduced 2.7-fold, 2.8-fold, 2.5-fold, and 2.7-fold in adipocytes treated with TNFα alone (0.62 ± 0.03, p < 0.001), the combination of TNFα, LPS, and PA (0.59 ± 0.05, p < 0.001), the combination of TNFα and PA (0.66 ± 0.05, p < 0.001), or the combination of TNFα and LPS (0.62 ± 0.02, p < 0.001), respectively (Fig. 4A). Similarly, *Sirt3* mRNA expression was also reduced 2.4-fold (0.74 ± 0.07, p < 0.01), 2.4-fold (0.74 ± 0.09, p < 0.01), 1.8-fold (0.97 ± 0.16, p < 0.05), and 2.1-fold (0.82 ± 0.03, p < 0.05) in adipocytes treated with either TNFα only, TNFα, LPS, and PA combination, TNFα and PA combination, or TNFα and LPS combination, respectively when compared to the control adipocytes (1.74 ± 0.30) (Fig. 4B). *Prkaa2* mRNA expression levels were significantly reduced in cells treated with TNFα only (2.0-fold, 0.79 ± 0.15, p < 0.05) compared to control cells (1.61 ± 0.17), while the other treatment conditions did not affect *Prkaa2* expression (Fig. 4C). The mRNA expression levels of *Cs*, *Sirt3*, and *Prkaa2* showed no significant difference in cells treated with TNFα only versus cells treated with the combination of TNFα, LPS, and PA (Fig. 4A–C).

### TNFα, LPS, and PA Differentially Regulate Endoplasmic Reticulum (ER) Stress-related Gene Markers in 3T3-L1 Adipocytes

**Fig. 5:**
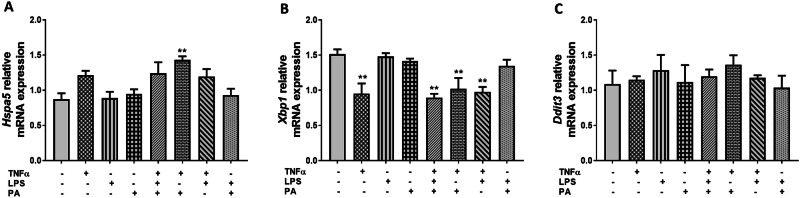
TNFα, LPS, and PA differentially regulates ER stress-related gene markers in 3T3-L1 adipocytes. Differentiated 3T3-L1 adipocytes were treated with TNFα (10 ng/mL), LPS (100 ng/mL), and PA (0.75 mM) individually or in combination for 24 h. The relative mRNA expression levels of *Hspa5* (**A**), *Xbp1* (**B**), and *Ddit3* (**C**) were measured using qRT-PCR. Results for *Hspa5*, *Xbp1*, and *Ddit3* mRNA are expressed relative to the average of *B2m* and *Rpl13a* as arbitrary units. Data are shown as the mean ± SEM of three independent biological experiments, each conducted in triplicate. Statistical significance between groups is denoted as **p < 0.01 compared to the untreated control adipocytes

qRT-PCR was used to quantify the mRNA expression of genes involved in ER stress in response to treatment with TNFα, LPS, and PA alone or in combination, including heat shock protein family A (Hsp70) member 5 (*Hspa5*), x-box binding protein 1 (*Xbp1*), and DNA-damage inducible transcript 3 (*Ddit3*). Compared to control adipocytes (0.87 ± 0.09), the mRNA expression levels of *Hspa5* were increased 1.6-fold (1.43 ± 0.05, p < 0.01) in adipocytes treated with TNFα and PA combination, while the 1.4-fold increase in *Hspa5* expression in adipocytes treated with TNFα individually, and the combination of TNFα, LPS, and PA or TNFα and LPS was not statistically significant (Fig. 5A). In contrast, *Xbp1* mRNA expression levels were reduced 1.6-fold (0.95 ± 0.15, p < 0.01), 1.7-fold (0.89 ± 0.06, p < 0.01), 1.5-fold (1.02 ± 0.16, p < 0.01), and 1.5-fold (0.98 ± 0.07, p < 0.01) in cells treated with TNFα only, or the combinations of TNFα, LPS, and PA, or TNFα and PA, or TNFα and LPS, respectively when compared to the untreated control cells (1.51 ± 0.07) (Fig. 5B). Furthermore, the mRNA expression of *Hspa5* and *Xbp1* was not significantly different between cells treated with TNFα only versus the combination treatments (Fig. 5A, B), while the mRNA expression levels of *Ddit3* remained unchanged in response to treatment with TNFα, LPS, and PA individually or in combination when compared to the untreated control cells (Fig. 5C).

### TNFα, LPS, and PA Combination Induce Oxidative Stress in 3T3-L1 Adipocytes

**Fig. 6:**
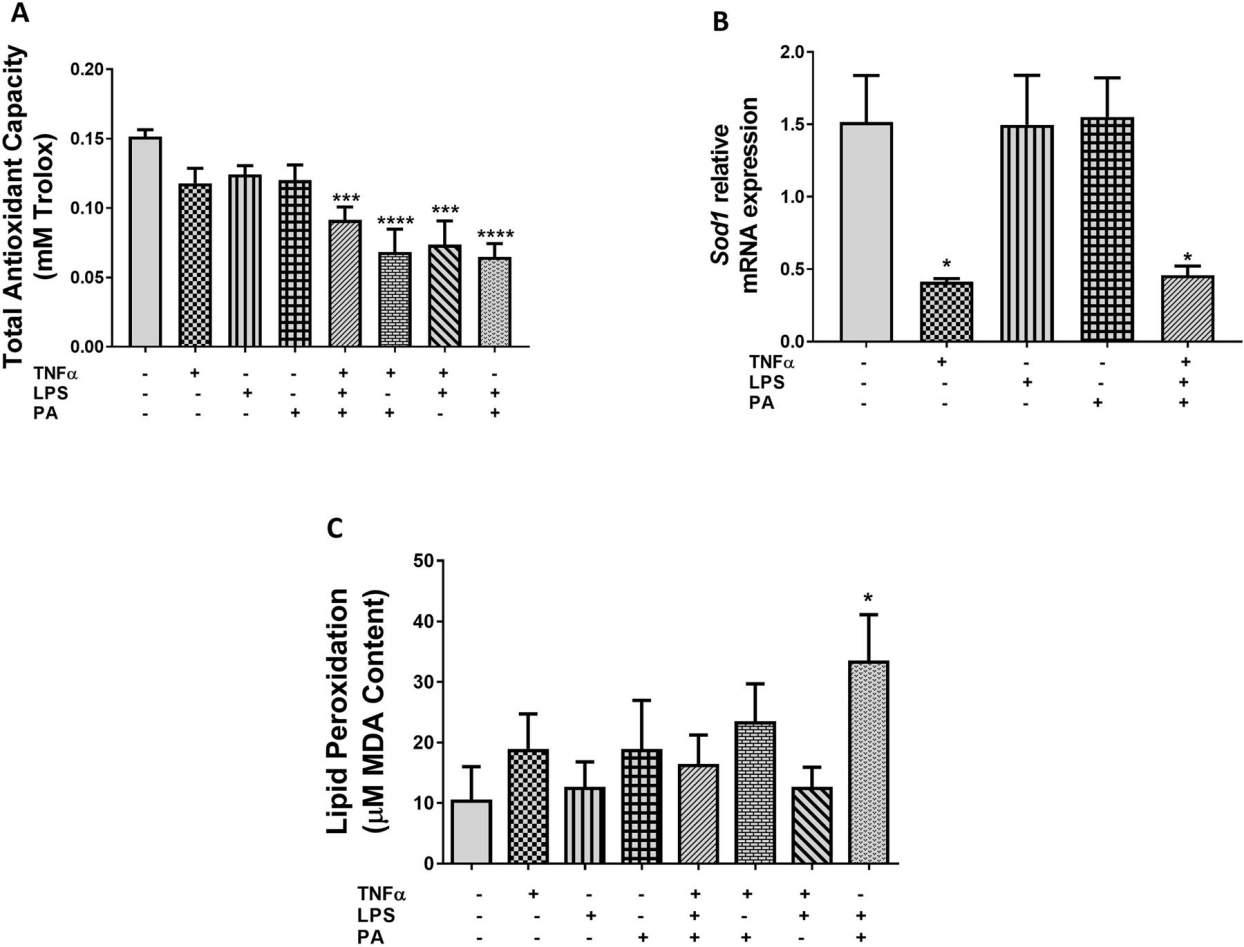
The combination of TNFα, LPS, and PA induces oxidative stress in 3T3-L1 adipocytes. Differentiated 3T3-L1 adipocytes were treated with TNFα (10 ng/mL), LPS (100 ng/mL), and PA (0.75 mM) individually or in combination for 24 h. Total antioxidant capacity (**A**) was assessed by quantifying antioxidant content using a commercial assay kit, the relative mRNA expression levels of *Sod1* (**B**) were measured using qRT-PCR, and lipid peroxidation (**C**) was assessed by quantifying MDA content using a commercial assay kit. Results for total antioxidant capacity and lipid peroxidation were expressed as a concentration derived from their standard curves, while *Sod1* mRNA was expressed relative to the average of *B2m* and *Rpl13a* as arbitrary units. Data are shown as the mean ± SEM of three independent biological experiments, each conducted in triplicate. Statistical significance between groups is denoted as *p < 0.05, ***p < 0.001, and ****p < 0.0001 compared to the untreated control adipocytes

The effect of TNFα, LPS, and PA alone or in combination on oxidative stress damage was assessed by quantifying the total antioxidant capacity and lipid peroxidation. Compared to control 3T3-L1 adipocytes (0.152 ± 0.005 mM), the antioxidant content was reduced 1.7-fold (0.091 ± 0.009 mM, p < 0.001), 2.2-fold (0.068 ± 0.017 mM, p < 0.0001), 2.1-fold (0.074 ± 0.017 mM, p < 0.001), and 2.3-fold (0.065 ± 0.009 mM, p < 0.0001) in cells treated with the combination of either TNFα, LPS, and PA, or TNFα and PA, or TNFα and LPS, or LPS and PA, respectively (Fig. 6A). The antioxidant capacity was also assessed by measuring *Sod1* mRNA expression and the results showed that treatment with TNFα only or the combination of TNFα, LPS, and PA reduced *Sod1* expression 3.7-fold (0.41 ± 0.02, p < 0.05) and 3.3-fold (0.46 ± 0.06, p < 0.05), respectively compared to the control cells (1.52 ± 0.32) (Fig. 6B). Lipid peroxidation was increased 3.2-fold (33.25 ± 7.85 µM, p < 0.05) in cells treated with the combination of LPS and PA when compared to the untreated control cells (10.33 ± 5.69 µM), while the other treatment conditions showed no statistical significance on lipid peroxidation when compared to the control (Fig. 6C).

### TNFα Downregulates the Expression Levels of Genes Involved in Insulin Signaling in 3T3-L1 Adipocytes

**Fig. 7:**
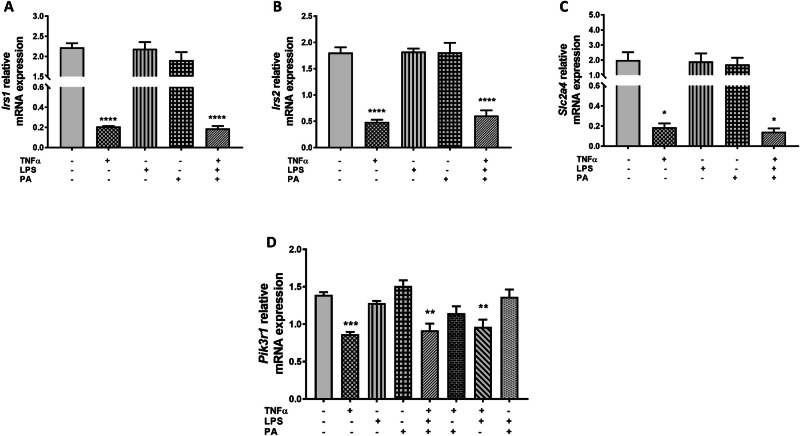
TNFα downregulates the expression levels of genes involved in insulin signaling in 3T3-L1 adipocytes. Differentiated 3T3-L1 adipocytes were treated with TNFα (10 ng/mL), LPS (100 ng/mL), and PA (0.75 mM) individually or in combination for 24 h. The relative mRNA expression levels of *Irs1* (**A**), *Irs2* (**B**), *Slc1a4* (**C**), and *Pik3r1* (**D**) were measured using qRT-PCR. Results for *Irs1*, *Irs2*, *Slc1a4*, and *Pik3r1* mRNA are expressed relative to the average of *B2m* and *Rpl13a* as arbitrary units. Data are shown as the mean ± SEM of three independent biological experiments, each conducted in triplicate. Statistical significance between groups is denoted as *p < 0.05, **p < 0.01, ***p < 0.001, and ****p < 0.0001 compared to the untreated control adipocytes

The effect of TNFα, LPS, and PA alone or in combination on insulin signaling was assessed by quantifying the mRNA expression levels of insulin receptor substrate 1 (*Irs1*), insulin receptor substrate 2 (*Irs2*), solute carrier family 2, facilitated glucose transporter member 4 (*Slc2a4*), and phosphoinositide 3 kinase regulatory subunit 1 (*Pik3r1*) using qRT-PCR. Compared to the control adipocytes (2.23 ± 0.10), the mRNA expression of *Irs1* was decreased 10.7-fold (0.21 ± 0.005, p < 0.0001) and 11.8-fold (0.19 ± 0.02, p < 0.0001) in adipocytes treated with TNFα only or the combination of TNFα, LPS, and PA, respectively (Fig. 7A). Similarly, *Irs2* was reduced 3.7-fold and 3.0-fold in adipocytes treated with TNFα individually (0.49 ± 0.04, p < 0.0001) or the combination of TNFα, LPS, and PA (0.61 ± 0.10, p < 0.0001) when compared to control adipocytes (1.81 ± 0.10) (Fig. 7B). The glucose transporter expression, *Slc2a4*, was also downregulated in cells treated with TNFα only (10.8-fold, 0.19 ± 0.04, p < 0.05) or the combination of TNFα, LPS, and PA (14.0-fold, 0.14 ± 0.03, p < 0.05) compared to control cells (2.00 ± 0.52) (Fig. 7C). Furthermore, mRNA expression levels of *Pik3r1* were reduced 1.6-fold, 1.5-fold, and 1.4-fold in adipocytes treated with TNFα individually (0.87 ± 0.03, p < 0.001), TNFα, LPS, and PA combination (0.92 ± 0.09, p < 0.01), and TNFα and LPS combination (0.96 ± 0.10, p < 0.01), respectively, compared to control adipocytes (1.39 ± 0.04) (Fig. 7D). The mRNA expression levels of *Irs1*, *Irs2*, *Slc2a4*, and *Pik3r1* showed no significant difference in cells treated with TNFα only versus cells treated with the combination treatments (Fig. 7A–D).

## Discussion

The murine 3T3-L1 adipocyte cell line, remains the most commonly used in vitro model for studying adipose tissue metabolism, the pathophysiology of adipocyte tissue dysfunction, identifying novel therapeutic targets, and screening potential therapeutic agents [33]. Previously, we showed that TNFα dysregulates lipid metabolism and induces inflammation in 3T3-L1 adipocytes, while treatment with LPS and PA has no effect, suggesting that TNFα treatment mimics an in vitro model of adipocyte dysfunction characterized by dysregulated lipid metabolism and increased inflammation [26]. In the current study, we conducted a comparative analysis to evaluate the effects of TNFα, LPS, and PA or their combination on mitochondrial dysfunction and associated metabolic complications including oxidative stress damage and insulin resistance in 3T3-L1 adipocytes.

We initially evaluated the effects of TNFα, LPS, and PA individually or in combination on the cells’ metabolic activity, mitochondrial function, and mitochondrial biogenesis. Overall, we demonstrate that TNFα, but not LPS or PA, has a detrimental impact on mitochondrial function in 3T3-L1 adipocytes by reducing intracellular ATP content, impairing mitochondrial respiratory function, decreasing mitochondrial content, and regulating the mRNA expression of related genes. In adipocytes, the mitochondria are mainly responsible for producing the majority of intracellular ATP through the process of mitochondrial respiration, which is required for various cell metabolic functions including triglyceride synthesis and lipolysis [34]. Our results showed reduced metabolic activity via decreased ATP content in TNFα-treated cells, and this is consistent with previous work demonstrating that TNFα induces mitochondrial dysfunction in 3T3-L1 adipocytes by reducing the production of intracellular ATP [35].

Our results also showed impaired mitochondrial respiration, as illustrated by decreased OCR in TNFα-treated cells. This data suggests that TNFα induces mitochondrial dysfunction by impairing mitochondrial OxPhos and shutting down the electron transport chain function as demonstrated by a reduction in basal respiration, ATP production, maximal respiration, and spare respiratory capacity in TNFα-treated cells. These results are in accordance with a previous study demonstrating that TNFα induces mitochondrial dysfunction by impairing mitochondrial respiration [36]. While TNFα treatments reduced OCR, ECAR was increased, suggesting that TNFα treatment increased the glycolytic activity in these cells, and this could be the means to balance the cellular energy demand of these cells. Impaired mitochondrial respiration is associated with reduced mitochondrial biogenesis. Our results showed significantly reduced *Ppargc1α* and *Nrf1* mRNA expression in TNFα-treated 3T3-L1 adipocytes. These genes are crucial for mitochondrial biogenesis [37]. Similar to our results, TNFα was previously shown to reduce *Ppargc1α* and *Nrf1* expression in 3T3-L1 adipocytes, while there was no significant difference in mtDNA copy number [35]. The lack of a notable change in mtDNA copy number suggests that TNFα treatment influences the quality and function of mitochondria rather than reducing their quantity.

Additionally, TNFα treatment led to decreased mRNA expression of key mitochondrial and metabolic regulators, including *Cs*, *Sirt3*, and *Prkaa2*. The reduced expression of these genes indicates the mechanism of TNFα-induced mitochondrial dysfunction, however, additional studies such as protein expression analysis are required to further elucidate the mechanism of TNFα-induced mitochondrial dysfunction. In obese in vivo models and individuals with obesity, the expression of *Cs*, *Sirt3*, and *Prkaa2* is reduced and is associated with mitochondrial dysfunction [38–40]. Thus, this suggests that in vitro treatment with TNFα in our study mimics the pathophysiology of obesity-related mitochondrial dysfunction. In contrast to TNFα, treatments with LPS, PA, or their combination did not affect the measured mitochondrial parameters. Although this highlights the unique role of TNFα in driving mitochondrial impairment, our results are not consistent with published literature. Several other studies have shown that treatment with LPS and PA induces mitochondrial dysfunction in 3T3-L1 adipocytes by reducing ATP content, impairing mitochondrial respiration, and reducing mitochondrial biogenesis [41–44]. These inconsistent results could be attributed to the differences in treatment conditions such as doses and durations, which were not standardized across the studies.

Impaired mitochondrial function is associated with endoplasmic reticulum (ER) stress and oxidative stress [45]. In this study, we showed that *Hspa5* expression was increased by treatment with a combination of TNFα and PA, thus suggesting increased ER stress. Consistently, results from other experimental models showed that the mRNA expression of *Hspa5*, also known as glucose-regulated protein 78 (*Grp78*) was increased in response to TNFα [46] and PA [47] treatments. Compared to TNFα and PA combination, treatment with TNFα and PA individually did not affect the mRNA expression of *Hspa5*, suggesting that TNFα and PA combination synergistically enhance ER stress beyond the levels induced by TNFα and PA alone. Unexpectedly, our results showed reduced *Xbp1* mRNA expression in TNFα-treated 3T3-L1 adipocytes. Contrary to our results, studies have shown that TNFα induces ER stress by increasing XBP1 expression [48, 49], however, these studies quantified spliced XBP1 protein expression, whose expression levels rapidly increase in response to ER stress compared to unspliced XBP1 [50]. The total antioxidant capacity and *Sod1* expression were reduced in TNFα-treated 3T3-L1 adipocytes, suggesting that TNFα induces oxidative stress in these cells by reducing their antioxidant capacity. These results are consistent with other studies demonstrating that TNFα treatment induces oxidative stress and decreases antioxidant capacity [51]. Interestingly, the combination of LPS and PA did lead to increased lipid peroxidation indicating that LPS and PA may contribute to oxidative stress only when combined.

Studies have shown that mitochondrial dysfunction induces insulin resistance in adipocytes via impaired insulin signaling pathways, which subsequently leads to reduced glucose uptake and the development of insulin resistance [52, 53]. Our results showed that TNFα treatments decreased the mRNA expression levels of *Irs1*, *Irs2*, *Scl2a4*, and *Pik3r1*, the key genes involved in insulin signaling. Our data aligns with previous research, supporting the notion that TNFα induces insulin resistance by impairing insulin signaling and reducing glucose uptake in adipocytes [35, 54]. Therefore, our results suggest that TNFα-induced mitochondrial dysfunction in 3T3-L1 adipocytes is associated with insulin resistance. Unlike previous data [55–59], our study showed that there was no effect on the total antioxidant capacity and the mRNA expression of *Sod1*, *Hspa5*, *Xbp1*, *Irs1*, *Irs2*, *Scl2a4*, and *Pik3r1* in response to treatment with LPS or PA, either individually or in combination. Once again, this could be due to the variation in the experimental conditions across these studies. In addition, this could also suggest that the cells activate compensatory mechanisms to mitigate the impact of LPS and PA treatments, but not the effects of TNFα treatment. As a limitation, this study did not directly quantify glucose uptake, and thus, the conclusions regarding the effect of TNFα on insulin resistance are solely based on mRNA expression data rather than direct functional assays. Another limitation of this study is the lack of protein expression analysis, which is crucial to confirm whether changes at the mRNA level translate to changes at the protein level and the functional outcomes. As such, further studies are warranted to evaluate the effects of TNFα treatment on the protein expression analysis.

In summary, our findings show that TNFα impairs mitochondrial function, induces oxidative stress, and disrupts insulin signaling in 3T3-L1 adipocytes. The combined treatment of TNFα, LPS, and PA produced similar effects as TNFα alone, indicating that TNFα is the primary factor driving these cellular and molecular alterations. These results highlight the significant role of TNFα in promoting mitochondrial dysfunction, oxidative stress, and insulin resistance, making it a promising target for the treatment of obesity-related metabolic disorders. Consequently, this study proposes that TNFα treatment can be utilized as a simple in vitro model to investigate the pathophysiology of mitochondrial dysfunction and associated metabolic complications such as oxidative stress and insulin resistance in adipocytes, and to screen potential anti-obesity therapeutics.
